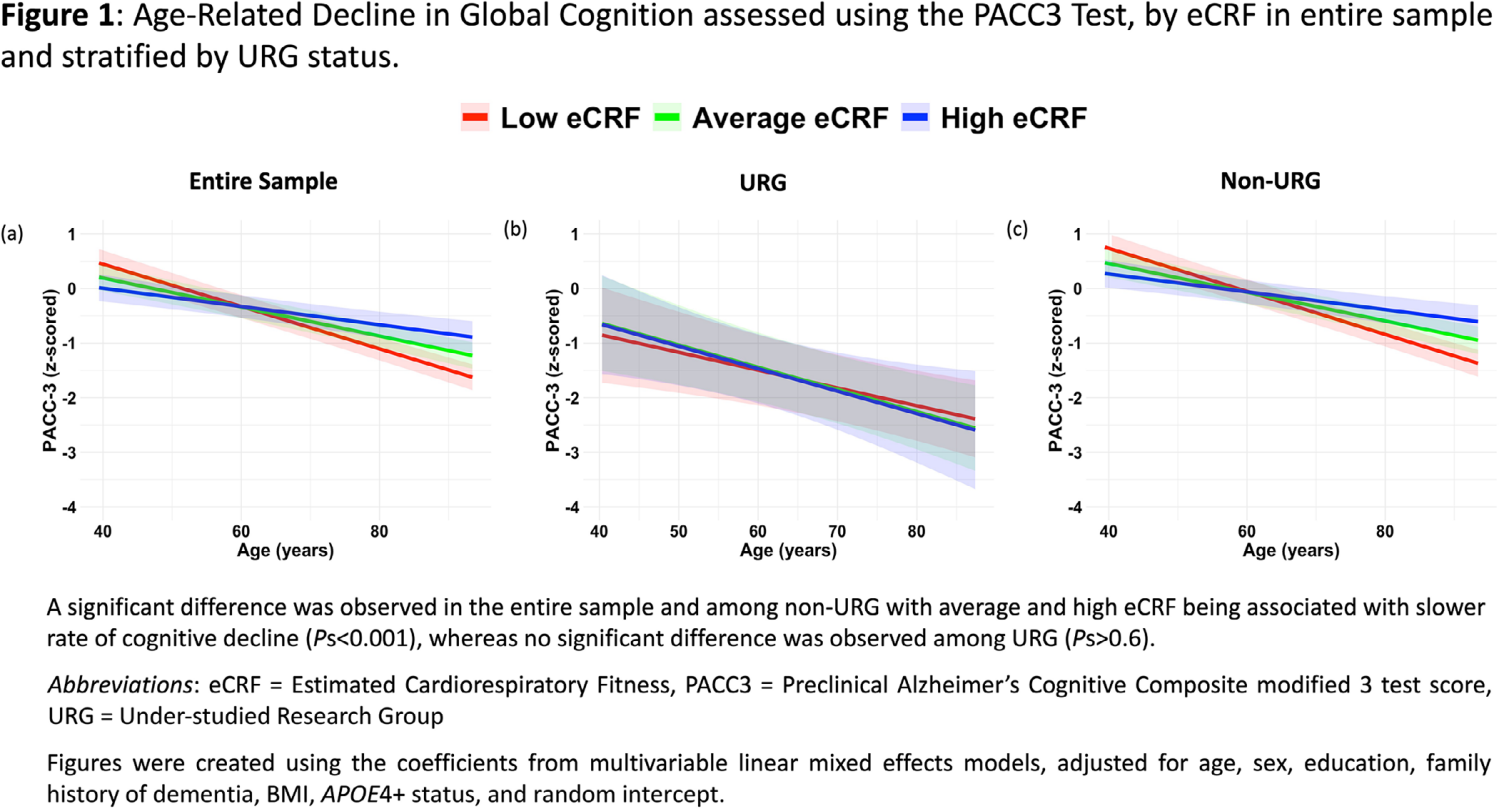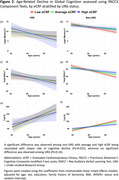# The Association of Cardiorespiratory Fitness with Rates of Cognitive Decline in a Multiethnic Cohort at Risk for Alzheimer’s Disease

**DOI:** 10.1002/alz70861_107989

**Published:** 2025-12-23

**Authors:** Ikenna S Odoh, Adam J Paulsen, Ira Driscoll, Brianne M. Breidenbach, Sarah R Lose, Bruce P Hermann, Sanjay Asthana, Sterling C Johnson, Dane B. Cook, Carey E. Gleason, Ozioma C. Okonkwo

**Affiliations:** ^1^ Wisconsin Alzheimer's Disease Research Center, School of Medicine and Public Health, University of Wisconsin‐Madison, Madison, WI USA; ^2^ Wisconsin Alzheimer's Disease Research Center, University of Wisconsin School of Medicine and Public Health, Madison, WI USA; ^3^ Department of Neurology, University of Wisconsin‐Madison School of Medicine and Public Health, Madison, WI USA; ^4^ Wisconsin Alzheimer's Institute, Madison, WI USA; ^5^ William S. Middleton Memorial Veterans Hospital, Madison, WI USA; ^6^ Department of Kinesiology, University of Wisconsin School of Education, Madison, WI USA; ^7^ Geriatric Research, Education and Clinical Center (GRECC), William S. Middleton Memorial Veterans Hospital, Madison, WI USA

## Abstract

**Background:**

Cardiorespiratory fitness (CRF) has emerged as a potential moderator of cognition. Higher CRF may offer cognitive resilience to individuals at risk for Alzheimer's Disease (AD). This, however, may vary across populations. Therefore, we aimed to examine whether the relationship between CRF and rates of cognitive decline differ for under‐studied research groups (URGs) compared to non‐URGs in a cohort of older cognitively unimpaired adults, enriched for AD risk.

**Methods:**

The sample consisted of 2006 participants [68% female; Mean_AGE_±SD=61.8±8.8 years; 12.5% URGs (74% African American, 26% others); Mean_FOLLOW‐UP_±SD=5.6±5.2 years] enrolled in the Wisconsin Registry for Alzheimer’s Prevention or the Wisconsin Alzheimer’s Disease Research Center. CRF was derived from a validated estimation equation (eCRF) and participants were categorized into tertiles (low, average and high) for analysis. Two‐sample t‐tests assessed cross‐sectional differences in eCRF between URGs and non‐URGs. Linear mixed effects models adjusting for age, sex, education, family history of dementia, BMI, and *APOE*4+ carrier status evaluated the association between eCRF and age‐related decline in global cognition, assessed by the Preclinical Alzheimer Cognitive Composite (PACC), in the entire sample and stratified by URG status. Secondary analyses were carried out on the three individual component tests of the PACC in stratified samples.

**Results:**

eCRF was significantly lower in URGs (*p* <0.001). Average and high eCRF was associated with slower decline in global cognition (*p*s <0.001)) across the entire sample. In stratified analyses, average and high eCRF were associated with slower decline in global cognition among non‐URGs (*p*s <0.001), but not URGs (*p*s >0.6). Secondary analyses of decline in performance on PACC’s three component tests revealed significant associations with average and high eCRF in non‐URGs (*p*s <0.015) but not URGs (*p*s >0.16).

**Conclusion:**

Average and high eCRF were associated with slower cognitive decline in non‐URGs, but not in URGs. These group differences may reflect underlying health or lifestyle disparities that influence fitness and cognition. Additionally, the eCRF measure used in this study was developed using data from a predominantly white population; therefore, may not accurately capture fitness status among URGs. This potential limitation should be addressed in future research to improve the accuracy and applicability of eCRF estimates in diverse populations.